# The characteristics and survival of second primary lung cancer after Hodgkin’s lymphoma: A comparison with first primary lung cancer using the SEER database

**DOI:** 10.1371/journal.pone.0285766

**Published:** 2023-05-17

**Authors:** Ling Lin, Daquan Wang, Haizhu Chen

**Affiliations:** 1 Department of Oncology, Tianjin Baodi Hospital, Baodi Clinical College of Tianjin Medical University, Tianjin, China; 2 Department of Radiation Oncology, Sun Yat-sen University Cancer Center, State Key Laboratory of Oncology in South China, Collaborative Innovation Center for Cancer Medicine, Guangzhou, Guangdong, China; 3 Breast Tumor Centre, Department of Medical Oncology, Phase I Clinical Trial Centre, Sun Yat-sen Memorial Hospital, Guangdong Provincial Key Laboratory of Malignant Tumor Epigenetics and Gene Regulation, Sun Yat-sen University, Guangzhou, China; Chung Shan Medical University, TAIWAN

## Abstract

**Objective:**

The study aimed to compare the characteristics and prognosis between patients with second primary lung cancer following Hodgkin’s lymphoma and those with primary lung cancer.

**Materials and methods:**

Using the SEER 18 database, the characteristics and prognosis were compared between the second primary non-small cell lung cancer following Hodgkin’s lymphoma (HL-NSCLC) (n = 466) and the first primary non-small cell lung cancer (n = 469,851)(NSCLC-1), as well as between the second primary small cell lung cancer following Hodgkin’s lymphoma (n = 93) (HL-SCLC) and the first primary small cell lung cancer (n = 94,168) (SCLC-1). Comparisons of categorical variables were performed using Chi-square or Fisher’s test. Continuous variables were compared using the Mann-Whitney U test. Overall survival (OS) was estimated using the Kaplan-Meier method, and the difference between groups was analyzed by log-rank test.

**Results:**

HL-NSCLC group had more males than NSCLC-1 group, and the median age of HL-NSCLC group was younger than that of NSCLC-1 group. Patients with HL-NSCLC showed inferior OS than those with NSCLC-1 (median: 10 months vs. 11 months, P = 0.006). Both HL-SCLC and SCLC-1 groups had poor prognosis, with median OS of 7 months (P = 0.4). The 3-year cumulative risks of death from any cause for patients with the latencies from HL to NSCLC of 0 to 5 years, >5 to 10 years, >10 to 15 years, >15 to 20 years, and>20 years were 71.8%, 82.6%, 86.8%, 85.7% and 78.5%, respectively(P = 0.020).

**Conclusion:**

HL-NSCLC patients had worse prognosis than NSCLC-1 patients, while HL-SCLC patients shared similar characteristics and survival with SCLC-1 patients.

## Introduction

Hodgkin’s lymphoma (HL) can be diagnosed at any age and it is most common in young adults [1]. Over the past decades, the remarkable progress in the treatment of HL has greatly improved survival outcomes, which makes HL one of the most curable cancers [2, 3]. With the combination of multi-agent chemotherapy and radiotherapy, the overall survival (OS) rates at 5 and 10 years for HL are 86% and 80%, respectively [1]. However, the prolonged survival following effective HL treatment has been accompanied by increased risk of subsequent malignancies, cardiovascular disease or other late effects [4–7]. According to the cancer databases from North America and Europe, approximately 6.6% to 12% of HL survivors developed second primary cancers during further follow-up, and lung cancer (LC) accounted for the most frequent type of solid tumors [6, 8–10]. A study from Sweden reported 2.39-fold increased risk of developing a second cancer among HL survivors, with a 3.3-fold increased risk of developing second lung cancer [9]. Lung cancer accounted for a large proportion of late deaths among HL survivors [11].

In previous studies, risk factors of developing second primary lung cancer following HL (HL-LC) have been explored, including older age at the time of HL diagnosis, male, time from HL treatment, alkylating chemotherapy, thoracic radiation, cigarette smoking and a family history of cancer [9, 11–14]. In contrast, data regarding the prognosis of HL-LC patients is very limited, and few studies have clarified the prognostic factors affecting survival outcomes. Several studies reported a median OS of 9 to 12.6 months for HL-LC patients, but their sample sizes were small and heterogeneity was high [15–17]. Additionally, the comparison of clinical characteristics and survival outcomes between HL-LC and the first primary lung cancer (LC-1) was extremely difficult, due to the relatively small number of HL-LC patients. Only one population-based study showed that patients with second primary non-small cell lung cancer (NSCLC) following HL (HL-NSCLC) had inferior OS compared to those with the first primary NSCLC(NSCLC-1) [18], probably due to molecular changes and limited treatment options influenced by prior therapy for HL [19]. Despite this, to date, no study has focused on the characteristics and prognosis of second primary small cell lung cancer (SCLC) following HL (HL-SCLC).

Therefore, in this study, the Surveillance, Epidemiology, and End Results (SEER) database(http://seer.cancer.gov/) was utilized to select all the patients with HL-NSCLC or HL-SCLC, as well as those with NSCLC-1 or the first primary SCLC (SCLC-1). We aimed to investigate the clinicopathological characteristics and survival outcomes of HL-LC patients, and compare them with LC-1 patients. We also explored the prognostic factors affecting OS among patients with HL-NSCLC or HL-SCLC.

## Materials and methods

### Study population

The SEER 18 Program database (Case Listing and Frequency Sessions: Incidence-SEER 18 Research Data + Hurricane Katrina Impacted Louisiana Cases, November 2018 Sub (1975–2016 varying)) (https://seer.cancer.gov/data-software/documentation/seerstat/nov2018) was used for patient collection in this study. This database contained the data of patients diagnosed between 1975 and 2016. Tumor histology was classified with the third edition of the International Classification of Diseases for Oncology (ICD-O-3). This study was approved by the Ethics Committee of National Cancer Institute (SEER Program), with an approval number of SAR0040750. This observational study used de-identified and publicly available data from SEER and thus did not require formal consent from patients.

**Fig 1:**
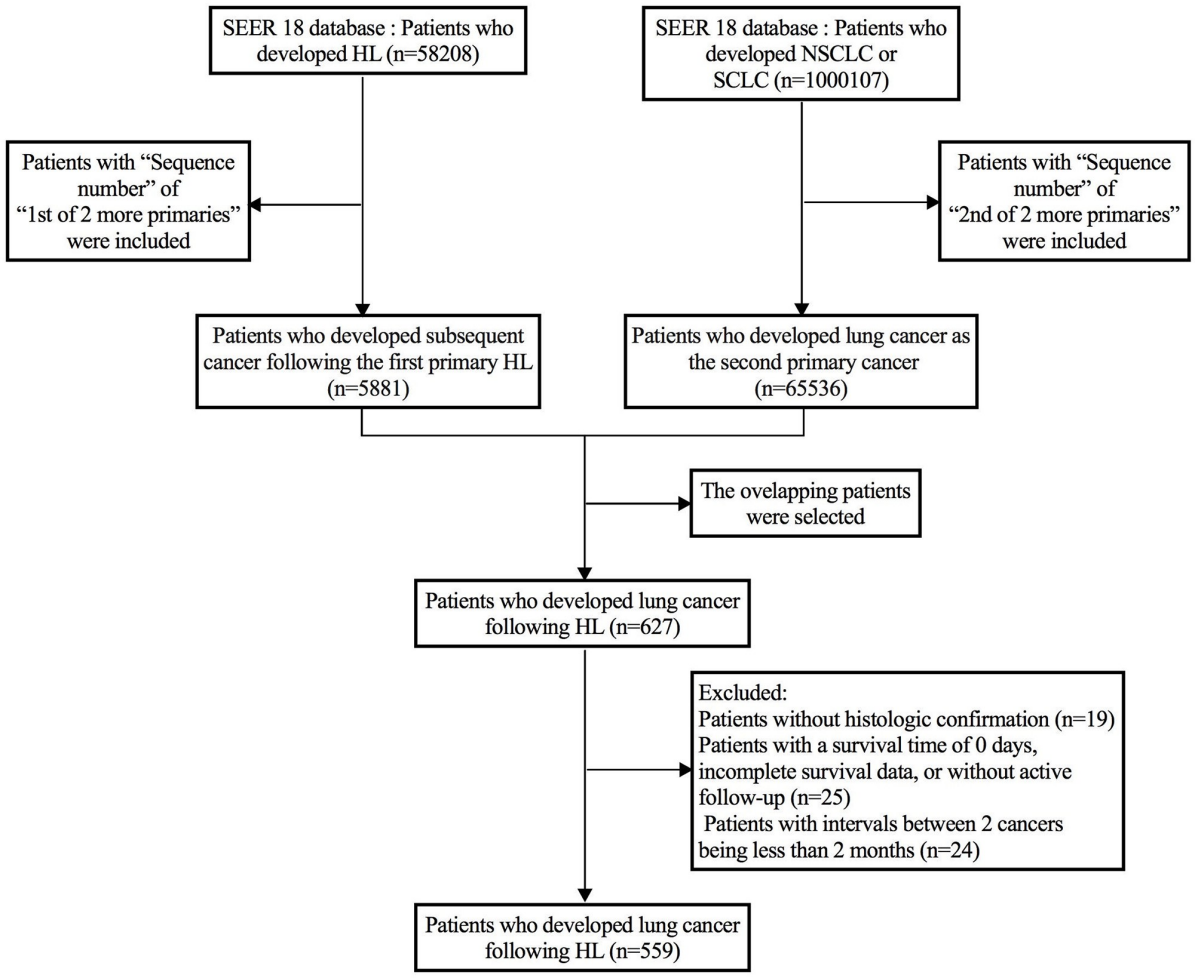
Patient selection flow.

Fig 1 showed the case selection diagram. Firstly, the ICD-O-3 histology codes of HL were used to select HL patients, including nodular sclerosis (9663, 9664, 9665, 9667), classical Hodgkin lymphoma (9650), lymphocyte-rich (9651), mixed cellularity (9652), lymphocyte-depleted (9653, 9655) and nodular lymphocyte predominant (9659). A total of 58208 patients with HL were identified after the first step. Secondly, the variable of “Sequence number” was collected for these HL patients, and only the patients with “Sequence number” of “1st of 2 or more primaries” were included. A total of 5881 patients who developed subsequent cancer following the first primary HL were identified after the second step.

Thirdly, the ICD-O-3 topographical codes (lung and bronchus (C34.0-C34.9)) and ICD-O-3 histology codes of LC (including squamous cell carcinoma (8070–8073, 8083), adenocarcinoma (8410, 8250–8253, 8255, 8260, 8323, 8480, 8481, 8550, 8560, 8570, 8574), large cell carcinoma (8012), NSCLC (8046) and SCLC (8041–8045)) were used to select LC patients. A total of 1000107 LC patients were identified after this step. Then, the variable of “Sequence number” was collected for these LC patients, and only the patients with “Sequence number” of “2nd of 2 or more primaries” were included. A total of 65536 patients who developed lung cancer as the second primary cancer were identified after the step.

The overlapping patients in the above two groups (5881 HL patients and 65536 LC patients) were selected. And a total of 627 patients who developed HL-LC were identified after the step. Forty-four patients with survival time of 0 days, with incomplete survival data, without active follow-up, or without histological confirmation were further excluded. A minimum latency of 2 months was required between initial HL and second primary lung cancer, in order to exclude synchronous primary cancers [20]. Thus twenty-four patients with interval between the two cancers being less than two months were excluded. Finally, a total of 559 patients were included for analysis, including 466 patients with HL-NSCLC and 93 patients with HL-SCLC.

Regarding the LC-1 group, a total of 1000107 primary lung cancer patients were collected from the same database. Of them, lung cancer was not the first primary malignancy in 213927 patients, and 141180 patients were diagnosed without histological confirmation. Therefore, they were excluded from the study. 83981 patients with a survival time of 0 days, with incomplete survival data, or without active follow-up were further excluded. Finally, a total of 564019 eligible patients were included for further analysis, consisting of 469851 NSCLC-1 and 94168 SCLC-1 patients.

### Retrieved variables and study endpoint

The demographics and survival data of patients were collected, including age at diagnosis, sex, year of diagnosis, race, grade, Ann Arbor stage, seer historic summary stage, laterality, survival time, survival status and causes of death. Treatment information, such as surgery, radiotherapy and chemotherapy, were also retrieved. Lung cancer patients were grouped into localized, regional or distant stage according to SEER historic summary stage. Details of this staging were described in historic SEER coding manuals (http://seer.cancer.gov/tools/codingmanuals/historical.html/). Generally, the neoplasm confined to the ipsilateral lung or bronchus was considered to be localized. The regional disease was defined by the involvement of hilar or mediastinal nodes and/or extension to regional structures. The neoplasm with metastatic spread beyond regional nodes or structures was classified as distant stage. The study endpoint of interest was OS. OS was measured from the date of lung cancer diagnosis to the date of death from any cause or last follow-up.

### Statistical analysis

Comparisons of categorical variables were performed using Chi-square or Fisher’s test. Continuous variables were compared using the Mann-Whitney U test. OS was estimated using the Kaplan-Meier method, and the difference between groups was examined by the log-rank test. The covariate-adjusted OS comparison between HL-LC and LC-1 groups was conducted based on a Cox proportional hazards model. Cox proportional hazard model was used to perform univariate and multivariate analysis for OS. The survival analysis was conducted among patients with complete lung cancer stage information. Variables with P values of less than 0.1 in the univariate analyses were included into the multivariate analysis. All analyses were two-sided, and *P*<0.05 was defined as statistically significant. All the analyses were done using SPSS software (version 26.0, SPSS, IBM) and R version 3.6.2 (http://www.r-project.org/).

## Results

### Patient characteristics at time of HL diagnosis

A total of 466 HL survivors developed NSCLC following HL, with a median latency of 10.3 years (range, 0.2–39.7 years), and 93 survivors developed SCLC following HL, with a median latency of 9.8 years (range, 0.34–35.1 years). Table 1 shows patient characteristics at time of HL diagnosis according to subsequent lung cancer stage. Patients with shorter latency from HL to lung cancer had earlier lung cancer stage, and the median latencies were 7, 10.1 and 15 years for localized, regional and distant stage groups, respectively(*P*<0.001). The age of patients at HL diagnosis was significantly different between the three stage groups, with median age of 55, 52 and 43 years for localized, regional and distant stage groups, respectively (*P*<0.001). Additionally, the HL patients diagnosed in 2000–2016 had earlier stage of lung cancer than those diagnosed in 1975–1999 (*P* = 0.002). In contrast, among the three stage groups for HL-SCLC, the distributions of latency, age, year at HL diagnosis and HL chemotherapy were similar.

**Table 1:** Patient characteristics at the time of HL diagnosis among HL-NSCLC and HL-SCLC.

Characteristic	Number of Patients With NSCLC					Number of Patients With SCLC				
	All	Localized	Regional	Distant	P	All	Localized	Regional	Distant	P
Total (n)	466	109	113	192		93	9	13	58	
Latency between HL and LC					**<0.001**					0.084
Median	10.3	7	10.1	15		9.8	11.8	8.2	10.8	
0–5	137(29.4)	44(40.4)	32(28.3)	32(16.7)		21(22.6)	1(11.1)	5(38.5)	10(17.2)	
>5–10	91(19.5)	21(19.3)	24(21.2)	32(16.7)		29(31.2)	2(22.2)	5(38.5)	15(25.9)	
>10–15	72(15.5)	17(15.6)	19(16.8)	33(17.2)		16(17.2)	5(55.6)	1(7.7)	10(17.2)	
>15–20	64(13.7)	9(8.3)	15(13.3)	39(20.3)		16(17.2)	0	2(15.4)	13(22.4)	
>20	102(21.9)	18(16.5)	23(20.4)	56(29.2)		11(11.8)	1(11.1)	0	10(17.2)	
Age at HL diagnosis					**<0.001**					0.835
Median	50	55	52	43		55	51	55	55	
0–39	136(29.2)	17(15.6)	29(25.7)	84(43.8)		15(16.1)	1(11.1)	2(15.4)	10(17.2)	
40–59	198(42.5)	47(43.1)	54(47.8)	69(35.9)		45(48.4)	5(55.6)	6(46.2)	28(48.3)	
60–69	89(19.1)	28(25.7)	20(17.7)	27(14.1)		25(26.9)	2(22.2)	4(30.8)	14(24.1)	
≥70	43(9.2)	17(15.6)	10(8.8)	12(6.3)		8(8.6)	1(11.1)	1(7.7)	6(10.3)	
Year of HL diagnosis					**<0.001**					0.554
1975–1999	319(68.5)	56(51.4)	71(62.8)	147(76.6)		73(78.5)	8(88.9)	9(69.2)	45(77.6)	
2000–2016	147(31.5)	53(48.6)	42(37.2)	45(23.4)		20(21.5)	1(11.1)	4(30.8)	13(22.4)	
HL subgroup					0.594					0.716
Nodular sclerosis	240(51.5)	49(45.0)	60(53.1)	108(56.3)		43(46.2)	3(33.3)	6(46.2)	27(46.6)	
Mixed cellularity	109(23.4)	36(33.0)	20(17.7)	37(19.3)		31(33.3)	5(55.6)	5(38.5)	18(31.0)	
Lymphocyte depleted	11(2.4)	1(0.9)	1(0.9)	5(2.6)		2(2.2)	0	1(7.7)	1(1.7)	
Lymphocyte rich	18(3.9)	5(4.6)	4(3.5)	8(4.2)		6(6.5)	0	0	5(8.6)	
Nodular lymphocyte predominant	11(2.4)	3(2.8)	3(2.7)	4(2.1)		0	0	0	0	
Classic, NOS	77(16.5)	15(13.8)	25(22.1)	30(15.6)		11(11.8)	1(11.1)	1(7.7)	7(12.1)	
Ann Arbor stage					0.659					0.671
I	97(20.8)	28(30.8)	27(31.0)	38(29.9)		19(20.4)	2(25.0)	3(27.3)	13(27.7)	
II	99(21.2)	27(29.7)	26(29.9)	43(33.9)		24(25.8)	3(37.5)	5(45.5)	16(34.0)	
III	65(13.9)	22(24.2)	21(24.1)	20(15.7)		15(16.1)	0	2(18.2)	12(25.5)	
IV	59(12.7)	14(15.4)	13(14.9)	26(20.5)		10(10.8)	3(37.5)	1(9.1)	6(12.8)	
Unknown	146(31.3)					25(26.9)				
HL Radiation					0.583					0.189
Yes	234(50.2)	53(49.5)	56(50.0)	104(56.8)		56(60.2)	6(66.7)	10(76.9)	30(51.7)	
No/unknown	232(49.8)	54(50.5)	56(50.0)	79(43.2)		37(39.8)	3(33.3)	3(23.1)	28(48.3)	
HL Chemotherapy					**0.016**					0.513
Yes	263(56.4)	69(63.3)	65(57.5)	90(46.9)		47(50.5)	5(55.6)	5(38.5)	35(60.3)	
No/unknown	203(43.6)	40(36.7)	48(42.5)	102(53.1)		46(49.5)	4(44.4)	8(61.5)	23(39.7)	

Note: *P* values represent Chi-square or Fisher’s test comparing the indicated variable distribution among localized, regional and distant patients. 52 HL-NSCLC and 13 HL-SCLC patients with unknown lung stage data were omitted from the calculation.

Abbreviation: HL, Hodgkin’s lymphoma; LC, lung cancer; HL-NSCLC, the second primary non-small lung cancer following Hodgkin’s lymphoma; NSCLC-1, the first primary non-small cell lung cancer; HL-SCLC, the second primary small lung cancer following Hodgkin’s lymphoma; SCLC-1, the first primary small cell lung cancer; NOS, not otherwise specified; NA, not available.

### Patient characteristics at time of lung cancer diagnosis

Table 2 summarized patient characteristics at the time of NSCLC diagnosis. The lung cancer stage was available in 414 patients, including 109 cases (26.3%) with localized stage, 113 (27.3%) with regional stage and 192 (46.3%) with distant stage. As for NSCLC-1, 387791 patients had complete stage information, in whom 86604 (22.3%), 113331 (29.2%) and 187856 (48.4%) patients had localized, regional and distant disease, respectively. Patients with HL-NSCLC were significantly younger than those with NSCLC-1, with median age of 61 (range, 23–87) and 67 (range, 5–105), respectively (*P*<0.001). Similar findings were observed among patients across three different stage groups. Males accounted for 65.5% in HL-NSCLC patients and 56.8% in NSCLC-1 patients(*P*<0.001). In the regional and distant stage groups, a larger proportion of patients received radiotherapy for NSCLC-1 patients, compared to the HL-NSCLC patients (regional, *P* = 0.014; distant, *P*<0.001).

**Table 2:** Patient characteristics at the time of NSCLC diagnosis.

Characteristic	All		Localized		Regional		Distant	
	HL-NSCLC	NSCLC-1	HL-NSCLC	NSCLC-1	HL-NSCLC	NSCLC-1	HL-NSCLC	NSCLC-1
Total	466	469,851	109	86604	113	113331	192	187856
Age at LC diagnosis, years								
Median(range), years	61(23–87)	67(5–105)	64(38–87)	70(10–103)	61(31–87)	68(9–104)	59(23–87)	67(5–105)
0–39	10(2.1)	3,891(0.8)	1(0.9)	300(0.3)	1(0.9)	652(0.6)	6(3.1)	2026(1.1)
40–49	69(14.8)	25,326(5.4)	12(11.0)	2640(3.0)	13(11.5)	5601(4.9)	38(19.8)	12,222(6.5)
50–59	121(26.0)	87,433(18.6)	22(20.2)	12,161(14.0)	33(29.2)	20,178(17.8)	53(27.6)	37,676(20.1)
60–69	144(30.9)	151,593(32.3)	31(28.4)	27,685(32.0)	38(33.6)	36,976(32.6)	56(29.2)	58,690(31.2)
≥70	122(26.2)	201,608(42.9)	43(39.4)	43,818(50.6)	28(24.8)	49,924(44.1)	39(20.3)	77,242(41.1)
*P*	**<0.001**		**<0.001**		**<0.001**		**<0.001**	
Sex								
Male	305(65.5)	266,879(56.8)	68(62.4)	43,209(49.9)	72(63.7)	64,637(57.0)	129(67.2)	107,497(57.2)
Female	161(34.5)	202,972(43.2)	41(37.6)	43,395(50.1)	41(36.3)	48,694(43.0)	63(32.8)	80,359(42.8)
*P*	**<0.001**		**<0.009**		0.151		**0.005**	
Race								
White	415(89.1)	377,344(80.3)	96(88.1)	72,366(83.6)	101(89.4)	91,929(81.1%)	172(89.6)	146,771(78.1)
Black	40(8.6)	59,360(12.6)	10(9.2)	8,651(10.0)	10(8.8)	13,854(12.2)	15(7.8)	24,789(13.7)
Others*	11(2.4)	33,147(7.1)	3(2.8)	5,587(6.5)	2(1.8)	7,548(6.7)	5(2.6)	15,296(8.1)
*P*	**<0.001**		0.266		**0.048**		**<0.001**	
Year of LC diagnosis								
1975–1999	120(25.8)	79,868(17.0)	17(15.6)	10,051(11.6)	26(23.0)	14,868(13.1)	37(19.3)	17,882(9.5)
2000–2016	346(74.2)	389,983(83.0)	92(84.4)	76,553(88.4)	87(77.0)	98,463(86.9)	155(80.7)	169,974(90.5)
*P*	**<0.001**		0.194		**0.002**		**<0.001**	
Histology								
SCC	155(33.3)	147,772(31.5)	51(46.8)	27,885(32.2)	46(40.7)	43,652(38.5)	34(17.7)	44,361(23.6)
Adenocarcinoma	238(51.1)	249,592(53.1)	49(45.0)	50,809(58.7)	48(42.5)	53,147(46.9)	119(62.0)	104,999(55.9)
LCC	22(4.7)	24,492(5.1)	1(0.9)	2,863(3.3)	5(4.4)	5376(4.7)	12(6.3)	9,395(5.0)
NSCLC, NOS	51(10.9)	47,995(10.2)	8(7.3)	5,047(5.8)	14(12.4)	11,156(9.8)	27(14.1)	29,101(15.5)
*P*	0.726		**0.005**		0.711		0.175	
Laterality								
Left	176(37.8)	187,001(39.8)	44(40.4)	35,675(41.2)	39(34.5)	46,288(40.8)	74(38.5)	71,877(38.3)
Right	266(57.1)	264,301(56.3)	65(59.6)	50,854(58.7)	72(63.7)	66,092(58.3)	102(53.1)	102,191(54.4)
Paired side	15(3.2)	16,846(3.6)	0(0.0)	24(0.0)	1(0.9)	726(0.6)	10(5.2)	12,606(6.7)
Unknown	9(1.9)	1703(0.4)	0(0.0)	51(0.1)	1(0.9)	225(0.2)	6(3.1)	1,182(0.6)
*P*	**<0.001**		0.930		0.133		**<0.001**	
Grade†								
I	31(6.7)	27,922(5.9)	16(14.7)	12,350(14.3)	5(4.4)	5,636(5.0)	6(3.1)	5,177(2.8)
II	99(21.2)	101,718(21.6)	35(32.1)	30,157(34.8)	31(27.4)	31,477(27.8)	24(12.5)	25,244(13.4)
III	138(29.6)	156,922(33.4)	28(25.7)	26,259(30.3)	38(33.6)	4,4607(39.4)	54(28.1)	60,677(32.3)
IV	18(3.9)	16,503(3.5)	2(1.8)	2084(2.4)	6(5.3)	3,677(3.2)	9(4.7)	5,447(2.9)
Unknown	180(38.6)	166,786(35.5)	28(25.7)	15,754(18.2)	33(29.2)	27,934(24.6)	99(51.6)	91,311(48.6)
*P*	0.424		0.340		0.486		0.446	
Surgery								
Yes	160(34.3)	157,169(33.5)	68(62.4)	61,285(70.8)	55(48.7)	54,822(48.4)	19(9.9)	14,137(7.5)
No	299(64.2)	302,871(64.5)	41(37.6)	24,824(28.7)	57(50.4)	57,841(51.0)	171(89.1)	17,2876(92.0)
Unknown	7(1.5)	9,811(2.1)	0(0.0)	495(0.6)	1(0.9)	668(0.6)	2(1.0)	843(0.4)
*P*	0.644		0.093		0.915		0.211	
Radiation								
Yes	152(32.6)	201,175(42.8)	26(24.1)	16,470(19.0)	41(36.6)	54,255(47.9)	70(36.5)	93,309(49.7)
No/unknown	314(67.4)	268,676(57.2)	82(75.9)	70,134(81.0)	71(63.4)	59,076(52.1)	122(63.5)	94,547(50.3)
*P*	**<0.001**		0.199		**0.014**		**<0.001**	
Chemotherapy								
Yes	190(40.8)	176,345(37.5)	15(13.8)	10,404(12.0)	61(54.0)	52,366(46.2)	108(56.2)	93,736(49.9)
No/unknown	276(59.2)	293,506(62.5)	94(86.2)	76,200(88.0)	52(46.0)	60,965(53.8)	84(43.8)	94,120(50.1)
*P*	0.149		0.575		0.097		0.780	

Note: Data are presented as n (%) expect where otherwise noted. *P* values represent Chi-square or Fisher’s test comparing the indicated variable distribution between HL-NSCLC and NSCLC-1 patients.

Abbreviation: LC, lung cancer; HL-NSCLC, the second primary non-small lung cancer following Hodgkin’s lymphoma; SCLC-1, the first primary non-small cell lung cancer; SCC, squamous cell carcinoma; LCC, large cell carcinoma; NOS, not otherwise specified.

*Others include Asian, Native American, Alaska Native/Pacific Islander.

^†^Grade I, II, III, IV refer to well differentiated, moderately differentiated, poorly differentiated and undifferentiated, respectively.

Regarding SCLC, the median age at LC diagnosis of HL-SCLC and SCLC-1 groups were 65 and 66 years, respectively (P = 0.135). There were 11.3% (n = 9), 16.3% (n = 13) and 72.5% (n = 58) of patients having localized, regional, and distant disease in the HL-SCLC group, and 5.8%, 23.3%, and 70.8% having localized, regional, and distant disease in the SCLC-1 group (*P* = 0.055). Fewer patients in the HL-SCLC group underwent radiotherapy compared to the SCLC-1 group (*P*<0.001). The detailed clinicopathologic features of patients with HL-SCLC and SCLC-1 at the time of SCLC diagnosis are displayed in Table 3.

**Table 3:** Patient characteristics at the time of SCLC diagnosis.

Characteristic	All patients		Patients with complete stage information	
	HL-SCLC	SCLC-1	HL-SCLC	SCLC-1
Total	93	94,168	80	75,751
Age at LC diagnosis, years				
Median(range), years	65(35–84)	66(15–99)	65(35–84)	67(15–99)
0–39	2(2.2)	485(0.5)	2(2.5)	373(0.5)
40–49	7(7.5)	4,725(5.0)	5(6.3)	3,707(4.9)
50–59	17(18.3)	19,590(20.8)	13(16.3)	15,299(20.2)
60–69	36(38.7)	33,239(35.3)	32(40.0)	26,328(34.8)
≥70	31(33.3)	36,129(38.4)	28(35.0)	30,044(39.7)
*P*	0.135		0.115	
Sex				
Male	50(53.8)	49,902(53.0)	43(53.8)	38,964(51.4)
Female	43(46.2)	44,266(47.0)	37(46.3)	36,787(48.6)
*P*	0.882		0.679	
Race				
White	86(92.5)	81,361(86.4)	73(91.3)	65,391(86.3)
Black	5(5.4)	8,864(9.4)	5(6.3)	7,023(9.3)
Others*	2(2.2)	3,943(4.2)	2(2.5)	3337(4.4)
*P*	0.231		0.434	
Year of LC diagnosis				
1975–1999	37(39.8)	14,066(14.9)	27(33.8)	8,259(10.9)
2000–2016	56(60.2)	80,102(85.1)	53(66.3)	67,492(89.1)
*P*	<0.001		<0.001	
Laterality				
Left	36(38.7)	37,741(40.1)	31(38.8)	30,093(39.7)
Right	49(52.7)	50,396(53.5)	42(52.5)	40,843(53.9)
Paired side	7(7.5)	5,345(5.7)	7(8.8)	4,209(5.6)
Unknown	1(1.1)	686(0.7)	0	606(0.8)
*P*	0.857		0.582	
Grade†				
I	0	141(0.1)	0	121(0.2)
II	0	518(0.6)	0	403(0.5)
III	8(8.6)	7,773(8.3)	8(10.0)	6,627(8.7)
IV	27(29.0)	30,905(32.8)	20(25.0)	22,633(29.9)
Unknown	58(62.4)	54,831(58.2)	52(65.0)	45,967(60.7)
P	0.851		0.763	
Stage				
Localized	9(9.7)	4,403(4.7)	9(11.3)	4,403(5.8)
Regional	13(14.0)	17,682(18.8)	13(16.3)	17,682(23.3)
Distant	58(62.4)	53,666(57.0)	58(72.5)	53,666(70.8)
Unknown	13(14.0)	18,417(19.6)	NA	NA
P	0.045		0.055	
Surgery				
Yes	7(7.5)	4,727(5.0)	6(7.5)	3,675(4.9)
No	85(91.4)	86,045(91.4)	74(92.5)	71,630(94.6)
Unknown	1(1.1)	3,396(3.6)	0	446(0.6)
P	0.260		0.557	
Radiation				
Yes	33(35.5)	42,625(45.3)	29(36.3)	34,054(45.0)
No/unknown	60(64.5)	51,543(54.7)	51(63.8)	41,697(55.0)
P	<0.001		0.118	
Chemotherapy				
Yes	65(69.9)	65,239(69.3)	58(72.5)	53,236(70.3)
No/unknown	28(30.1)	289,29(30.7)	22(27.5)	22,515(29.7)
P	0.898		0.664	

Note: Data are presented as n (%) expect where otherwise noted. *P* values represent Chi-square or Fisher’s test comparing the indicated variable distribution between HL-SCLC and SCLC-1 patients

Abbreviation: LC, lung cancer; HL-SCLC, the second primary small lung cancer following Hodgkin’s lymphoma; SCLC-1, the first primary small cell lung cancer; NA, not available.

*Others include Asian, Native American, Alaska Native/Pacific Islander.

^†^Grade I, II, III, IV refer to well differentiated, moderately differentiated, poorly differentiated and undifferentiated, respectively.

### Overall survival of HL-LC versus LC-1

**Fig 2:**
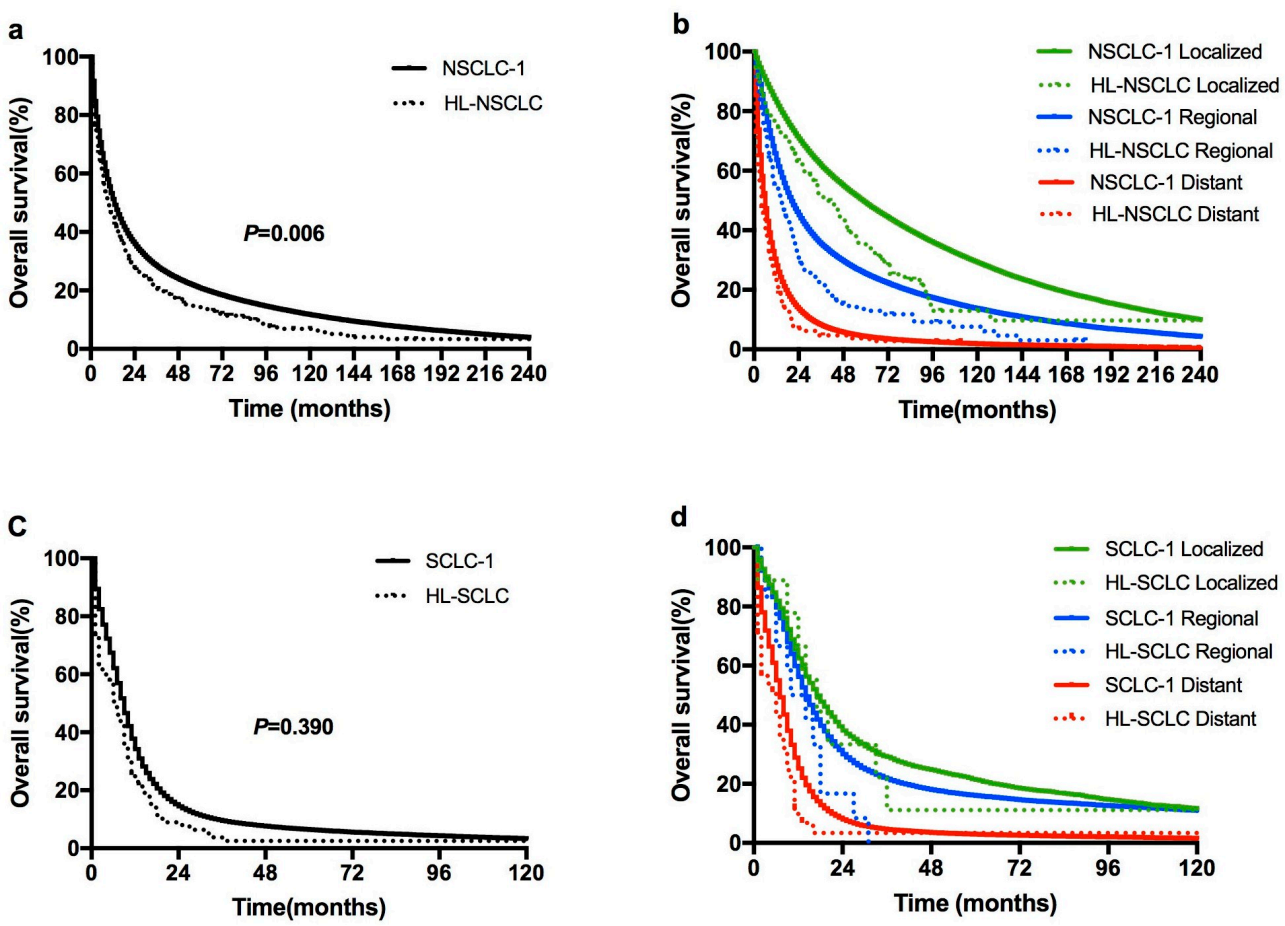
Kaplan-Meier estimates of OS. (a) Comparison of OS between HL-NSCLC and NSCLC-1; (b) Comparison of OS between HL-NSCLC and NSCLC-1 according to lung cancer stage; (c) Comparison of OS between HL-SCLC and SCLC-1; (d) Comparison of OS between HL-SCLC and SCLC-1 according to lung cancer stage.

Table 4 summarizes the OS of HL-NSCLC versus NSCLC-1, and HL-SCLC versus SCLC-1. For all patients with NSCLC, the OS of HL-NSCLC group was significantly inferior than that of NSCLC-1 group, with the median OS of 10 and 11 months, respectively (*P* = 0.006) (Fig 2a). Similar findings were found in both localized and regional disease groups (*P* = 0.003 and 0.002, respectively), whereas no difference was observed between the HL-NSCLC and NSCLC-1 groups in patients with distant disease (*P* = 0.200) (Fig 2b). As for SCLC, the median OS were both 7 months for HL-SCLC and SCLC-1 patients (Fig 2c and 2d).

**Table 4:** Comparison of overall survival between HL-LC and LC-1.

	Non-small cell lung cancer		Small cell lung cancer	
	HL-NSCLC	NSCLC-1	HL-SCLC	SCLC-1
All patients	n = 466	n = 392954	n = 93	n = 94168
6-month	59.6	61.8	50.5	52.6
1-year	44.9	47.3	22.9	29.0
2-year	27.4	33.1	8.9	12.2
5-year	14.1	19.2	2.6	5.4
Median(95%CI), months	10(8–12)	11(11–11)	7(4–9)	7(7–7)
P*	**0.006**		0.400	
HR(95%CI), *P*†	1.272(1.152–1.404), P<**0.001**		1.188(0.961–1.470), *P* = 0.112	
Localized	n = 109	n = 86604	n = 9	n = 4403
6-month	81.0	88.5	88.9	77.9
1-year	74.9	81.1	66.7	59.3
2-year	63.3	69.3	33.3	36.2
5-year	35.3	48.2	11.1	20.3
Median(95%CI),months	39(30–52)	56(56–57)	17(12-NA)	16(15–17)
P*	0.003		0.500	
HR(95%CI), *P*†	1.445(1.149–1.818), P = **0.002**		0.918(0.457–1.843), *P* = 0.809	
Regional	n = 113	n = 113331	n = 13	n = 17682
6-month	71.6	76.3	66.7	71.9
1-year	53.5	61.2	50	49.6
2-year	30.5	43.4	16.7	25.4
5-year	12.9	24.4	0	11.8
Median(95%CI),months	14(10–20)	19(19–19)	12(6-NA)	12(12–13)
P*	**0.002**		0.300	
HR(95%CI), *P*†	1.400(1.151–1.703), *P* = **0.001**		1.150(0.653–2.028), *P* = 0.628	
Distant	n = 192	n = 187856	n = 58	n = 53666
6-month	40.4	41.5	43.4	44.4
1-year	22.7	24.7	9.1	20.4
2-year	7.0	11.9	3.4	6.4
5-year	3.7	3.8	3.4	2.3
Median(95%CI),months	4(3–6)	5(5–5)	6(2–810.004)	5(5–5)
P*	0.200		0.300	
HR(95%CI), *P*†	1.177(1.013–1.367), P = **0.033**		1.131(0.861–1.484), *P* = 0.376	

Note: Data are presented as percentages expect where otherwise noted.

Abbreviation: HL-LC, the second primary lung cancer following Hodgkin’s lymphoma; LC-1, the first primary lung cancer; HL-NSCLC, the second primary non-small lung cancer following Hodgkin’s lymphoma; NSCLC-1, the first primary non-small cell lung cancer; HL-SCLC, the second primary small lung cancer following Hodgkin’s lymphoma; SCLC-1, the first primary small cell lung cancer; HR, hazard ratio; CI, confidential interval; NA, not avaliable

*P values were calculated from a 2-tailed log-rank test comparing HL-NSCLC with NSCLC-1, or HL-SCLC with SCLC-1

^†^HRs and *P* values were HL-NSCLC versus NSCLC-1, or HL-SCLC versus SCLC-1, and obtained from a Cox proportional hazards model that included history of HL, age at LC diagnosis, sex, race, year of LC diagnosis, LC subtype (not included in the model for SCLC), laterality, LC grade, LC stage, surgery for LC, radiotherapy and chemotherapy for LC. Patients with NSCLC-1 or SCLC-1 was chosen as the reference group.

Covariate-adjusted OS comparison between HL-NSCLC and NSCLC-1 groups was further conducted, based on a Cox proportional hazards model that included history of HL, age at LC diagnosis, sex, race, year of LC diagnosis, LC subtype, laterality, LC grade, LC stage, surgery for LC, radiotherapy and chemotherapy for LC (Table 4). After adjusting these variables, HL-NSCLC patients consistently had inferior OS compared with NSCLC-1 patients in both localized and regional groups (localized, hazard ratio [HR] 1.445, 95% confidence interval [CI] 1.149–1.818, *P* = 0.003; regional, HR 1.400, 95%CI 1.151–1.703, *P* = 0.002). Importantly, in the distant stage group, HL-NSCLC patients also showed significantly worse OS than NSCLC-1 patients (HR 1.177, 95%CI 1.013–1.367, *P* = 0.033). No difference in OS was observed between HL-SCLC and SCLC-1 groups (Table 4).

### Death causes of HL-LC versus LC-1

**Fig 3:**
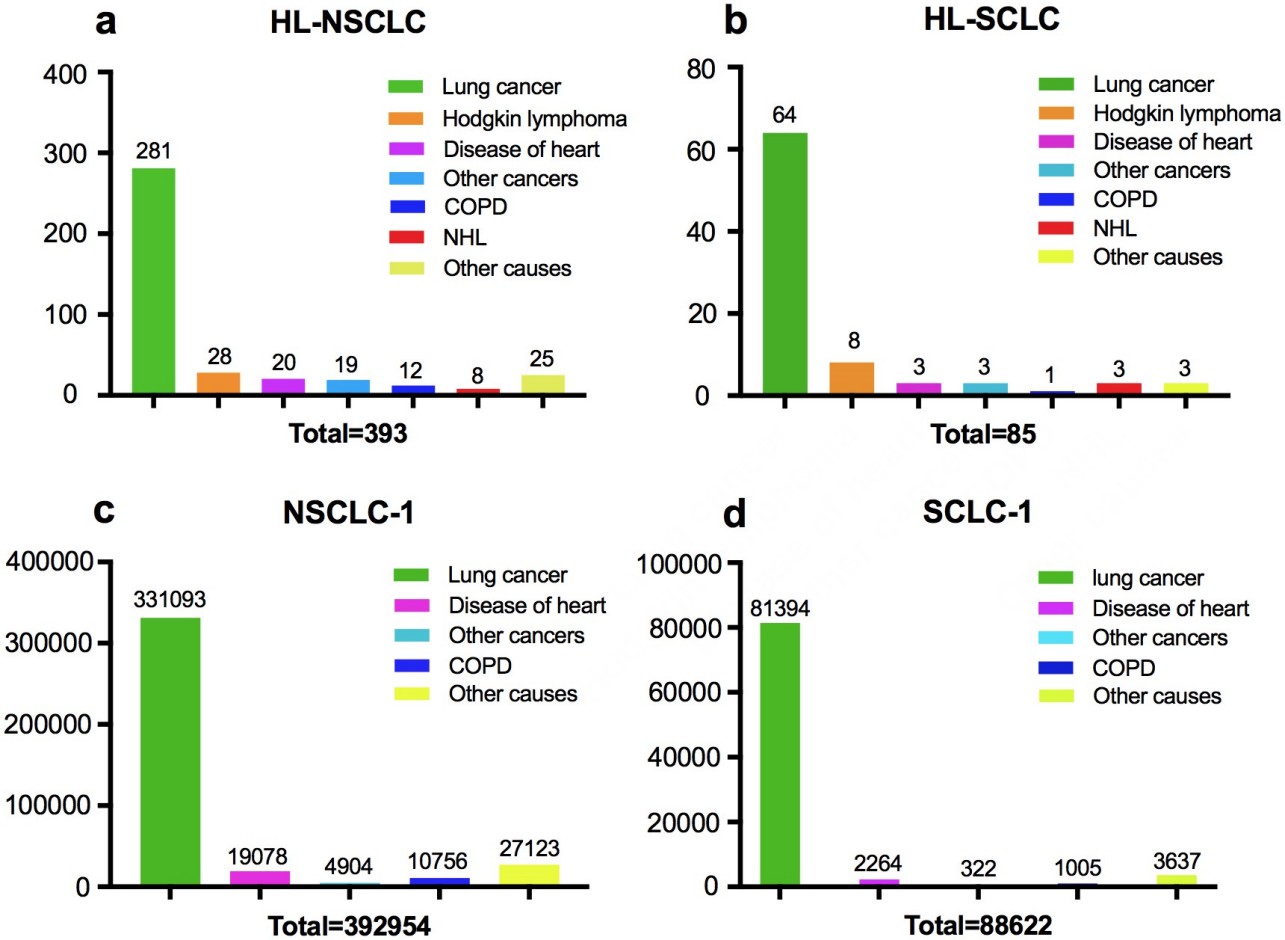
Causes of deaths. (a) Causes of deaths among HL-NSCLC; (b) Causes of deaths among HL-SCLC; (c) Causes of deaths among NSCLC-1; (d) Causes of deaths among SCLC-1.

A total of 393 (84.3%) and 85 (91.4%) deaths were recorded among HL-NSCLC and HL-SCLC patients, respectively. The death causes grouped by histology of lung cancer were shown in Fig 3. Lung cancer accounted for the first leading cause of death for both HL-NSCLC (281/393, 71.5%) and HL-SCLC (64/85, 75.3%), followed by HL [(28/393, 7.1%) and (8/85, 9.4%), respectively], disease of heart [(20/393, 5.1%) and (3/85, 3.5%), respectively], and other cancers [(19/393, 4.8%) and (3/85, 3.5%), respectively]. Likewise, for patients with NSCLC-1 or SCLC-1, lung cancer accounted for the most common cause of death [(331093/392954, 84.3%) and (81394/88622, 91.8%), respectively]. The percentage of death from other cancers in the LC-1 group was lower than that in the HL-LC group. Besides, compared to HL-LC patients, fewer patients in the LC-1 group died from non-Hodgkin’s lymphoma.

### Prognostic factors among HL-NSCLC

Table 5 summarized the results of univariate and multivariate analysis among 414 HL-NSCLC patients with lung cancer stage information available. The univariate analysis showed that sex, year of HL diagnosis, HL subtype, Ann arbor stage, chemotherapy for HL, year of lung cancer diagnosis, LC subtype, laterality, LC stage, LC grade, surgery for LC, and the latency were factors affecting OS.

**Table 5:** Univariate and multivariate analysis of OS in patients with HL-NSCLC.

Characteristic	No.	Univariate analysis		Multivariate analysis	
		HR(95%CI)	P	HR(95%CI)	P
Age at HL diagnosis					
0–39	130	Reference		ND	ND
40–59	170	0.851(0.661–1.094)	0.208	ND	ND
60–69	75	0.828(0.609–1.126)	0.23	ND	ND
≥70	39	0.802(0.531–1.210)	0.292	ND	ND
Year of HL diagnosis					
1975–1999	274	Reference		Reference	
2000–2016	140	0.765(0.608–0.963)	0.022	1.175(0.605–2.281)	0.634
Sex					
Male	269	Reference		Reference	
Female	145	0.772(0.616–0.968)	0.025	0.599(0.283–0.949)	0.029
Race					
White	369	Reference		ND	ND
Black	35	0.838(0.572–1.227)	0.364	ND	ND
Other	10	1.200(0.617–2.332)	0.591	ND	ND
HL subtype					
Nodular sclerosis	217	Reference		Reference	
Mixed cellularity	93	0.771(0.589–1.007)	0.057	0.905(0.534–1.533)	0.905
Lymphocyte depleted	7	2.276(1.006–5.151)	0.048	1.328(0.192–9.164)	0.774
Lymphocyte rich	17	0.796(0.470–1.347)	0.395	1.351(0.545–3.352)	0.516
Classic, NOS	70	0.775(0.574–1.046)	0.096	0.690(0.418–1.136)	0.145
Nodular lymphocyte predominant	10	1.061(0.522–2.157)	0.87	1.070(0.259–4.427)	0.926
Ann Arbor stage					
I	93	Reference		Reference	
II	96	1.32(0.961–1.812)	0.086	1.447(0.869–2.408)	0.156
III	63	0.936(0.648–1.353)	0.726	1.226(0.693–2.170)	0.483
IV	53	1.470(1.022–2.113)	0.038	1.386(0.764–2.516)	0.282
HL Radiation					
Yes	213	Reference		ND	ND
No	189	0.862(0.695–1.069)	0.176	ND	ND
HL Chemotherapy					
Yes	224	Reference		Reference	
No	190	1.395(1.129–1.725)	0.002	0.923(0.582–1.465)	0.735
Age at lung cancer diagnosis					
20–49	71	Reference		ND	ND
50–59	108	1.173(0.841–1.634)	0.347	ND	ND
60–69	125	1.066(0.772–1.471)	0.699	ND	ND
≥70	110	0.979(0.705–1.361)	0.901	ND	ND
Year of lung cancer diagnosis					
1977–1999	80	Reference		Reference	
2000–2016	334	0.653(0.507–0.842)	0.001	0.68(0.3–1.539)	0.355
LC subtype					
Squamous cell carcinoma	131	Reference		Reference	
Adenocarcinoma	216	1.171(0.92–1.49)	0.199	0.736(0.476–1.137)	0.167
Large cell carcinoma	18	1.821(1.104–3.004)	0.019	2.058(0.337–12.583)	0.434
NSCLC, NOS	49	1.991(1.402–2.829)	<0.001	1.970(0.922–4.213)	0.08
Laterality					
Left	157	Reference		Reference	
Right	239	0.946(0.758–1.180)	0.623	0.968(0.646–1.452)	0.876
Paired side	11	3.824(1.994–7.334)	<0.001	NA	0.703
LC stage					
Localized	109	Reference		Reference	
Regional	113	1.808(1.334–2.451)	<0.001	1.853(1.166–2.944)	0.009
Distant	192	3.978(2.975–5.319)	<0.001	4.277(2.336–7.829)	<0.001
Grade					
I	27	Reference		Reference	
II	90	1.753(0.984–3.124)	0.057	1.226(0.571–2.628)	0.601
III	120	2.583(1.474–4.527)	0.001	1.527(0.727–3.211)	0.264
IV	17	4.621(2.257–9.461)	<0.001	0.82(0.145–4.646)	0.822
LC surgery					
Yes	142	Reference		Reference	
No	269	2.541(2.008–3.216)	<0.001	1.740(1.048–2.888)	0.032
Radiation					
Yes	137	Reference		ND	ND
No	273	0.877(0.698–1.101)	0.257	ND	ND
Chemotherapy					
Yes	184	Reference		ND	ND
No	230	1.067(0.862–1.320)	0.553	ND	ND
Latency					
0–5	108	Reference		Reference	
>5–10	77	1.293(0.942–1.775)	0.112	0.836(0.493–1.417)	0.506
>10–15	69	1.414(1.013–1.975)	0.042	0.896(0.447–1.796)	0.757
>15–20	63	1.594(1.139–2.230)	0.007	0.664(0.240–1.836)	0.43
>20	97	1.115(0.829–1.518)	0.49	0.772(0.278–1.876)	0.504

Abbreviation: OS, overall survival; HL-NSCLC, the second primary non-small lung cancer following Hodgkin’s lymphoma; HL, Hodgkin’s lymphoma, LC, lung cancer; HR, hazard ratio; CI, confidential interval; NOS, not otherwise specified; ND, not done.

*Others include Asian, Native American, Alaska Native/Pacific Islander.

^†^Grade I, II, III, IV refer to well differentiated, moderately differentiated, poorly differentiated and undifferentiated, respectively.

The survival analysis was conducted among patients with complete lung cancer stage information.

After multivariate analysis, sex (*P* = 0.029), LC stage (regional vs. localized, *P* = 0.009; distant vs. localized, *P*<0.001) and surgery for LC (*P*<0.032) were independent factors predicting OS.

### Prognostic factors among HL-SCLC

The survival analysis was conducted in 80 HL-SCLC patients with lung cancer stage information available. As shown in Table 6, the factors affecting OS examined by univariate analysis included age at LC diagnosis, LC stage, and chemotherapy for LC. Multivariate analysis showed that age at LC diagnosis (60–69 vs. 0–49, HR 4.669, 95%CI 1.39–15.681, *P* = 0.013; ≥70 vs. 0–49, HR 3.657, 95%CI 1.070–12.494, *P* = 0.039), LC stage (distant vs. localized, HR 4.362, 95%CI 1.784–10.667, *P* = 0.001) and chemotherapy for LC (No/unknown vs. yes, HR 2.302, 95%CI 1.218–4.351, *P* = 0.01) were independent factors affecting OS.

**Table 6:** Univariate and multivariate analysis of OS in patients with HL-SCLC.

Characteristic	No.	Univariate analysis		Multivariate analysis	
		HR(95%CI)	P	HR(95%CI)	P
Age at HL diagnosis					
0–39	13	Reference		ND	ND
40–59	39	0.718(0.365–1.414)	0.338	ND	ND
60–69	20	0.998(0.480–2.074)	0.995	ND	ND
≥70	8	1.621(0.658–3.996)	0.294	ND	ND
Year of HL diagnosis					
1975–1999	62	Reference		ND	ND
2000–2016	18	0.801(0.430–1.494)	0.485	ND	ND
Sex					
Male	43	Reference		ND	ND
Female	37	0.911(0.570–1.456)	0.697	ND	ND
Race					
White	73	Reference		ND	ND
Black	5	0.489(0.152–1.574)	0.23	ND	ND
Hlsubgroup					
Nodular sclerosis	36	Reference		ND	ND
Mixed cellularity	28	0.895(0.538–1.488)	0.669	ND	ND
Lymphocyte depleted	2	1.287(0.173–9.554)	0.805	ND	ND
Lymphocyte rich	5	0.743(0.262–2.112)	0.578	ND	ND
Classic, NOS	9	1.410(0.581–3.421)	0.447	ND	ND
Ann Arbor stage					
I	18	Reference		ND	ND
II	24	1.122(0.587–2.142)	0.728	ND	ND
III	14	1.699(0.790–3.652)	0.175	ND	ND
IV	10	0.891(0.388–2.046)	0.786	ND	ND
HL Radiation					
Yes	46	Reference		ND	ND
No	34	1.344(0.831–2.173)	0.228	ND	ND
HL Chemotherapy					
Yes	45	Reference		ND	ND
No	35	0.909(0.567–1.457)	0.691	ND	ND
Age at lung cancer diagnosis					
20–49	7	Reference		Reference	
50–59	13	2.178(0.74–6.404)	0.157	3.53(0.926–13.455)	0.065
60–69	32	3.203(1.220–8.408)	0.018	4.669(1.39–15.681)	0.013
≥70	28	3.193(1.205–8.461)	0.02	3.657(1.070–12.494)	0.039
Year of lung cancer diagnosis					
1977–1999	27	Reference		ND	ND
2000–2016	53	0.995(0.614–1.615)	0.985	ND	ND
Laterality					
Left	31	Reference		Reference	
Right	42	1.597(0.971–2.624)	0.065	1.465(0.854–2.513)	0.165
Paired side	7	1.154(0.472–2.819)	0.754	0.755(0.225–2.533)	0.649
LC stage					
Localized	9	Reference		Reference	
Regional	13	1.672(0.673–4.155)	0.268	1.421(0.531–3.803)	0.484
Distant	58	3.663(1.652–8.120)	0.001	4.362(1.784–10.667)	0.001
LC surgery					
Yes	6	Reference		ND	ND
No	74	1.874(0.745–4.717)	0.182	ND	ND
Radiation					
Yes	29	Reference		ND	ND
No	51	1.478(0.902–2.422)	0.121	ND	ND
Chemotherapy					
Yes	58	Reference		Reference	
No	22	1.674(0.997–2.808)	0.05	2.302(1.218–4.351)	0.01
Latency					
0–5	16	Reference		ND	ND
>5–10	22	1.460(0.716–2.975)	0.298	ND	ND
>10–15	16	1.323(0.613–2.854)	0.475	ND	ND
>15–20	15	1.930(0.882–4.225)	0.1	ND	ND
>20	11	2.158(0.909–5.120)	0.081	ND	ND

Abbreviation: OS, overall survival; HL-SCLC, the second primary small lung cancer following Hodgkin’s lymphoma; HL, Hodgkin’s lymphoma, LC, lung cancer; HR, hazard ratio; CI, confidential interval; NOS, not otherwise specified; ND, not done.

*Others include Asian, Native American, Alaska Native/Pacific Islander.

^†^Grade I, II, III, IV refer to well differentiated, moderately differentiated, poorly differentiated and undifferentiated, respectively.

The survival analysis was conducted among patients with complete lung cancer stage information.

### The cumulative risk of death affected by the latency between diagnosis of HL and LC

**Fig 4:**
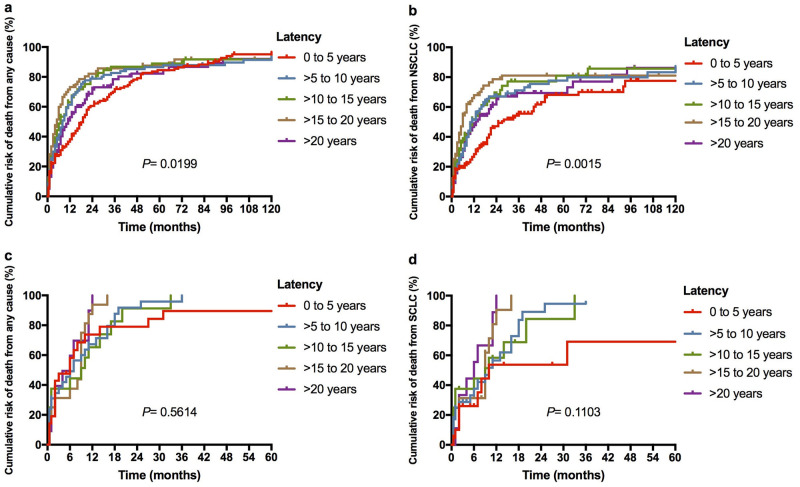
The cumulative risk of death based on the latency between diagnoses of HL and LC. (a) The cumulative risk of death from any cause based on the latency between diagnoses of HL and NSCLC; (b) The cumulative risk of death from NSCLC based on the latency between diagnoses of HL and NSCLC; (c) The cumulative risk of death from any cause based on the latency between diagnoses of HL and SCLC; (d) The cumulative risk of death from SCLC based on the latency between diagnoses of HL and SCLC.

The mortality risks affected by the latency between diagnosis of HL and LC were analyzed. Notably, the 3-year cumulative risks of death from any cause for HL-NSCLC patients with latencies of 0–5 years, >5 to 10 years, >10 to 15 years, >15 to 20 years, and>20 years were 71.8%, 82.6%, 86.8%, 85.7% and 78.5%, respectively(P = 0.020) (Fig 4a). Likewise, the cumulative risk of death from NSCLC was also affected by the latency, and the 3-year cumulative risks were 55.6%, 71.2%, 77.1%, 81.0% and 69.4% for the above five latency groups, respectively (P = 0.002) (Fig 4b). As for patients with HL-SCLC, the latency between HL and SCLC showed no effect on the cumulative risk of death from either any cause (P = 0.561) or SCLC (P = 0.110) (Fig 4c and 4d).

## Discussion

Due to the rarity of HL-LC, it is difficult to thoroughly investigate this disease entity in large series. Until now, few studies have reported the characteristics and outcomes of HL-LC. In this large population-based study, we performed a direct comparison of the characteristics and survival between the HL-LC group and the LC-1 group. The survival outcomes of HL-SCLC were described for the first time. We demonstrated that HL-NSCLC patients had an inferior OS compared with NSCLC-1 patients (*P* = 0.006), whereas no significant difference in OS was observed between HL-SCLC patients and SCLC-1 patients (*P* = 0.390). Besides, this study explored the prognostic factors affecting OS for HL-NSCLC and HL-SCLC, which might provide guidance for monitoring and treating these patients.

According to a systematic review conducted by Lorigan et al., HL survivors had an increased risk of 9.7% for developing second lung cancer, and the risk increased with time from initial treatment of HL, for as long as 20–25 years [11]. Our study revealed a median latency of 10 years between diagnosis of HL and lung cancer. Besides, 21.9% and 11.8% of patients developed HL-NSCLC and HL-SCLC with a long latency of more than 20 years, respectively. These data indicated the importance of long-term regular medical examinations for HL survivors. The chest computed tomography (CT) should be performed to ensure the early detection of second primary lung cancer [21–23]. Besides, our study also indicated that patients with shorter latency, older age and HL diagnosed in a more recent year tended to have earlier stage of secondary NSCLC. There were several possible explanations for these findings. Firstly, more frequent examinations might be performed in the first five years after treatment for HL patients, which led to a large proportion of cases incidentally diagnosed. Secondly, patients with older age were more likely to undergo medical examinations for pertinent symptoms compared to those with younger age. Furthermore, the increased health awareness of people, as well as the improved diagnostic techniques, might promote early detection and diagnosis of cancer.

The second primary NSCLC might have different biological characteristics compared with the first primary NSCLC, which could be examined by next-generation sequencing [24]. Prior radiotherapy or chemotherapy against HL could lead to the occurrence of genetic alterations in patients, which had an effect on the biologic behavior of the second primary lung cancer. One previous study compared the tumor tissues between patients with the first primary NSCLC and those with the second primary NSCLC after radiation for HL, and the results indicated that HL-NSCLC patients had increased number of microsatellite alterations [19]. Our study observed an inferior OS for HL-NSCLC compared to NSCLC-1, which might be partly due to the biological changes caused by prior treatment against HL.

For patients with HL-LC, the prior treatment against HL might also cause injury of normal organs (especially for the heart, lung and bone marrow), thus reducing the tolerance to treatment against lung cancer. The OS was affected not only by the deaths from specific cancer but also other causes such as complications and treatment side effects. Therefore, compared to cancer specific survival (CSS), OS was a more sensitive endpoint to examine the difference in prognosis between HL-LC and LC-1 patients. Furthermore, the quality of OS was more reliable than that of CSS, theoretically. Based on the above reasons, the OS was chosen as the endpoint of interest in the study. It should be noted that, the difference in OS between HL-NSCLC and NSCLC in this study could be also due to the higher risk of comorbidities and treatment side effects for HL-NSCLC patients.

SCLC accounts for 15–20% of lung cancer cases, which showed more aggressive behavior than NSCLC. Schoenfeld et al. identified 55 patients with secondary lung cancer from 1976 primary HL patients, and only 11 patients belonged to HL-SCLC [17]. Another study reported 27 patients with secondary lung cancer following HL, and only 2 patients were diagnosed with HL-SCLC [15]. No study has reported the survival outcomes of HL-SCLC, due to the very limited sample size. To the best of our knowledge, we conducted the first population-based study to report the characteristics and survival of HL-SCLC patients. HL-SCLC accounted for 16.7% of all HL-LC patients. Besides, HL-SCLC patients shared no difference with SCLC-1 patients in terms of age, sex, race and stages. Most HL-SCLC patients (72.5%) presented with distant stage at the time of SCLC diagnosis, which was similar to SCLC-1 patients (70.8%), reflecting the highly aggressive nature of SCLC. Fewer HL-SCLC patients received radiotherapy compared with SCLC-1 patients, and the reason might lie in prior radiotherapy against the primary HL. Both patients with HL-SCLC and SCLC-1 had poor survival outcomes, with median OS of 7 months. It remains unclear whether HL-SCLC had genetic alterations in tumor tissue compared with SCLC-1 patients, and further studies are warranted to explore that.

Despite that lung cancer was the first leading cause of death for both HL-LC and LC-1 patients, other causes of death were slightly different between the two groups. Firstly, quite a few patients with HL-LC died of Hodgkin’s lymphoma, which indicated the importance of management of primary HL disease. Besides, this study showed that a higher proportion of patients in the HL-LC group died from non-Hodgkin’s lymphoma (NHL) compared with those in LC-1 groups, which was consistent with previous study. Petrakova et al. reported that the NHL was one of the most common secondary cancers among primary HL survivors, with the standardized incidence ratio of 13.1 [25].

Few studies have explored the prognostic factors affecting the survival outcome of HL-LC. In this study, based on multivariate analysis, the variables of sex, LC stage and LC surgery remained the independent factors affecting OS. Females had superior OS than males, which was consistent with previous studies involving primary NSCLC [26, 27]. It was not surprising that patients with localized stage and those receiving surgery obtained superior OS than those not, since they had limited tumor burden and good performance status. Among patients with HL-SCLC, older age and not receiving chemotherapy for LC were correlated with a worse OS. It might be due to the poor tolerance to treatment for these patients.

There were several limitations in this study. First, several important variables are not available in SEER database, such as the performance status, smoking history, medical comorbidities, radiation dose-fractionation schedules, the systemic therapy information and treatment response, etc. The radiation and chemotherapy information was displayed as "Yes" or "No/unknown". "No" and "unknown" were grouped together and further detailed information was not available. Therefore, interpretation of data is limited. Second, the SEER historic stage, instead of TNM stage was used for analysis in the study, because it is the only consistent staging system available in the database throughout the years. Thirdly, since the OS was chosen as the endpoint of interest in this study, it could not be determined whether the difference in prognosis between HL-LC and LC-1 was due to cancer specific death or other reasons, and it required further investigations. Despite these limitations, this study included a large cohort of HL-LC patients with long follow-up time, which allowed for analyses of survival outcomes and death causes.

## Conclusion

In conclusion, patients who developed second primary lung cancer after Hodgkin’s lymphoma had worse prognosis than those with primary lung cancer. The percentage of deaths from lung cancer in the HL-NSCLC group was lower than that in the NSCLC-1 group. However, patients with HL-SCLC patients shared similar characteristics and survival outcomes with SCLC-1 patients. Further studies are warranted to elucidate the molecular biology of HL-LC patients.

## Abbreviations

CI: confidential interval
CT: computed tomography
HL: Hodgkin’s lymphoma
HL-LC: the second primary lung cancer following Hodgkin’s lymphoma
HL-NSCLC: the second primary non-small cell lung cancer following Hodgkin’s lymphoma
HL-SCLC: the second primary small cell lung cancer following Hodgkin’s lymphoma
HR: hazard ratio
ICD-O-3: the third edition of the International Classification of Diseases for Oncology
LC: lung cancer
LC-1: the first primary lung cancer
NHL: non-Hodgkin’s lymphoma
NSCLC: non-small cell lung cancer
NSCLC-1: the first primary non-small cell lung cancer
OS: overall survival
SCC: squamous cell carcinoma
SCLC: small cell lung cancer
SCLC-1: the first primary small cell lung cancer
SEER: Surveillance, Epidemiology, and End Results
